# Mechanisms of Oocyte Maturation and Related Epigenetic Regulation

**DOI:** 10.3389/fcell.2021.654028

**Published:** 2021-03-19

**Authors:** Meina He, Tuo Zhang, Yi Yang, Chao Wang

**Affiliations:** ^1^Department of Biology, School of Basic Medical Science, Guizhou Medical University, Guiyang, China; ^2^Department of Physiology, School of Basic Medical Sciences, Guizhou Medical University, Guiyang, China; ^3^State Key Laboratory of Agrobiotechnology, College of Biological Sciences, China Agricultural University, Beijing, China; ^4^Key Laboratory of Ministry of Education for Conservation and Utilization of Special Biological Resources in the Western China, College of Life Science, Ningxia University, Yinchuan, China

**Keywords:** ovary, oocyte, meiosis arrest, meiosis resumption, oocyte maturation

## Abstract

Meiosis is the basis of sexual reproduction. In female mammals, meiosis of oocytes starts before birth and sustains at the dictyate stage of meiotic prophase I before gonadotropins-induced ovulation happens. Once meiosis gets started, the oocytes undergo the leptotene, zygotene, and pachytene stages, and then arrest at the dictyate stage. During each estrus cycle in mammals, or menstrual cycle in humans, a small portion of oocytes within preovulatory follicles may resume meiosis. It is crucial for females to supply high quality mature oocytes for sustaining fertility, which is generally achieved by fine-tuning oocyte meiotic arrest and resumption progression. Anything that disturbs the process may result in failure of oogenesis and seriously affect both the fertility and the health of females. Therefore, uncovering the regulatory network of oocyte meiosis progression illuminates not only how the foundations of mammalian reproduction are laid, but how mis-regulation of these steps result in infertility. In order to provide an overview of the recently uncovered cellular and molecular mechanism during oocyte maturation, especially epigenetic modification, the progress of the regulatory network of oocyte meiosis progression including meiosis arrest and meiosis resumption induced by gonadotropins is summarized. Then, advances in the epigenetic aspects, such as histone acetylation, phosphorylation, methylation, glycosylation, ubiquitination, and SUMOylation related to the quality of oocyte maturation are reviewed.

## Introduction

Uncovering the signals involved in controlling the resumption of oocyte meiosis is a major issue in female reproductive biology. The meiosis initiation and resumption of oocytes is different from sperm in at least three aspects. Female germ cells enter and undergo the first meiotic progression during embryonic development, and arrest at the diplotene stage of prophase I before birth. And, some of the arrested oocytes within fully grown follicles will resume meiosis after puberty in response to luteinizing hormones (LHs) during each estrous (animal) or menstrual cycle (human) (Mehlmann, 2005). Last, the cell division of oocytes is known as asymmetric cytokinesis. Interestingly, whenever fully grown oocytes are released from follicles and cultured in appropriate medium *in vitro*, spontaneous resumption happens as well (Pincus and Enzmann, 1935). Oocyte meiotic maturation is a complicated and vital process used to attain full competence required for the oocyte as well as early embryonic development. An oocyte arrested at meiotic prophase I contains a large nucleus covered by a nuclear envelope, which is known as the germinal vesicle (GV). With the arrival of LH surge, serial processes related to oocyte nuclear maturation, such as chromatin condensation and germinal vesicle breakdown (GVBD), occur in oocytes of fully grown follicles. After GVBD, oocytes enter the metaphase I (MI) stage (Moor et al., 1998). Later, after extrusion of the first polar body (PB1) containing a small portion of cytoplasm, an oocyte containing one set of chromosomes completes meiosis I. Very soon after that, the second meiosis starts and the oocyte (mature egg) arrests at metaphase II (MII) until fertilization. Actually, the oocyte accomplishes its meiosis progress only when fertilization happens.

In humans and animals, multiple factors including epigenetic molecules and different signaling pathways have been identified and proven to be pivotal for meiotic maturation. They not only regulate oocytes maturation, but also coordinate with each other to ensure good oocyte quality. This article aims to review the events and development around the quality control of mammalian oocyte meiotic maturation in nuclear and cytoplasm aspects, of which, the underlying molecular mechanisms are discussed to provide detailed information for better understanding of meiosis.

## Oocyte Nuclear Maturation

### The Regulation Mechanism of Oocyte Meiosis Arrest at Prophase I

Before an oocyte is enclosed by ovarian granulosa cells to form primordial follicles, meiosis has been initiated and the cell has arrested at the diplotene stage of prophase I (Bowles et al., 2006; Bowles and Koopman, 2007). When females are sexually mature, a small portion of primordial follicles will be activated and start to grow gradually. Previous studies have indicated that molecules such as cyclic adenosine monophosphate (cAMP) within growing oocytes and the natriuretic peptide precursor type C (NPPC)/natriuretic peptide receptor 2 (NPR2) system in granulosa cells play essential roles in maintaining oocyte meiotic arrest during the long developmental journey. Later, oocytes in fully grown follicles in response to gonadotropins stimulation possess the capability to resume meiosis and ovulate *in vivo*.

#### High cAMP Level Within Oocyte Contributes to Meiotic Arrest

In mammals, meiotic arrest is regulated by a high level of cAMP in the oocyte (Conti et al., 2002; Mehlmann, 2005). When oocytes are isolated from the antral follicles, the cAMP levels within the oocytes decrease and meiosis resumes spontaneously (Törnell et al., 1990). On the contrary, when they are cultured with the cAMP analog dibutyryl cAMP (dbcAMP) or cAMP phosphodiesterase (PDE) inhibitors such as isobutyl methyl xanthine (IBMX) and milrinone, the spontaneous meiotic maturation of mouse oocytes is prevented (Cho et al., 1974; Dekel et al., 1981; Schultz et al., 1983; Vivarelli et al., 1983; Eppig et al., 1985; Aktas et al., 1995). Therefore, a constantly higher level of cAMP becomes the priority for oocytes to sustain meiosis at the GV stage.

cAMP in oocytes plays a central role in the regulation of meiosis arrest (Zhang et al., 2009). Oocytes possess all of the necessary proteins including adenylyl cyclase (AC), Gs protein, and G protein-coupled receptor 3 (GPR3) for producing cAMP themselves. AC is responsible for specifically catalyzes ATP to form cAMP, and Gs protein, which stimulates AC3 activity in oocytes (Horner et al., 2003; Hinckley et al., 2005; Mehlmann, 2005). Mice oocytes lacking AC3 expression fail to maintain meiosis arrest (Horner et al., 2003). Similarly, blocking Gs function causes spontaneous resumption of meiosis in follicle-enclosed mouse oocytes (Mehlmann et al., 2002; Kalinowski et al., 2004). GPR3, which is located in the oocyte plasma membrane, is necessary to stimulate Gs activity and elevate the level of cAMP (Kalinowski et al., 2004). This is approved by the fact that oocytes undergo spontaneous meiotic resumption at an early antral stage in *GPR3* KO mice and the phenomenon can be reversed by injection of *GPR3* mRNA into the oocyte (Freudzon, 2005). The studies in pig oocytes are consistent with those in mice (Yang et al., 2012a). Although GPR3 is expressed in the human oocyte, it contributes nothing to premature ovarian failure, which is unlike the phenotype of *GPR3* KO mice (Kovanci et al., 2008). While GPR and Gs are functional in generating intrinsic cAMP, PDE in mice oocytes is responsible for the degradation of cAMP (Sasseville et al., 2006). In a *PDE3* knockout model, oocytes are permanently arrested at the GV stage and female mice are infertile (Vaccari et al., 2008). Specifically, inhibition of PDE3 elevates cAMP level and prevents oocyte spontaneous maturation simultaneously in cultured cumulus-oocyte-complexes (COCs) or denuded oocytes (DOs) (Kovanci et al., 2008). Simultaneously knockout of *GPR3* and *PDE3A* result in oocyte maturation (Vaccari et al., 2008).

#### NPPC/NPR2 System in Granulosa Cells Contributes to Meiotic Arrest

Meiosis inhibition is a process in which oocytes coordinate with granulosa cells to sustain a high level of cAMP. Cumulative data have proven that intrinsic cAMP produced by oocyte alone is not sufficient to maintain meiotic arrest. Instead, a sustained high level of cAMP in the oocyte depends on cGMP, which is produced in the surrounding granulosa cells, possibly by suppressing PDE3A activity (Zhang et al., 2010; Shuhaibar et al., 2015; Jaffe and Egbert, 2017). Generally, cGMP is produced from GTP by guanylyl cyclases in mural granulosa cells (MGCs) and cumulus granulosa cells (CGCs) and is transported to oocytes.

cGMP production in CGCs relies on the coordination of MGCs-secreted NPPC conjugating with its receptor, guanylyl cyclase NPR2 which is found on the membrane of CGCs. NPPC and NPR2 are both highly expressed in follicular granulosa cells (Zhang et al., 2010; Jaffe and Egbert, 2017). NPPC inhibits the spontaneous GVBD in COCs, but not in DOs *in vitro*. Besides, *NPR2* mutant mice are infertile due to premature resumption of meiosis because of the shortage of cGMP production in CGCs, which results in oocyte fragmentation and poor embryo development (Geister et al., 2012; Tsuji et al., 2012). Consistently, applying NPPC in cultured COCs contributes to preventing spontaneous oocyte maturation by increasing the cGMP levels in the CGCs (Zhang et al., 2010). Together, these results suggest that cGMP produced in granulosa cells play a vital role in keeping the cAMP level high in the oocyte, and that maintaining oocyte meiotic arrest requires coordination between granulosa cells and an oocyte within a follicle.

How is the NPPC/NPR2 signaling pathway regulated in granulosa cells? One of the important actions of follicle stimulation hormones (FSHs) on MGCs and CGCs of antral follicles is to sustain high levels of NPPC/NPR2 in humans, rodents, and pig (Jankowski et al., 1997; Kawamura et al., 2011). Pregnant mare serum gonadotrophin (PMSG) that possesses primarily FSH activity induces the expression of *NPPC* and *NPR2* mRNA in the ovary (Zhang and Xia, 2012). This is further approved by the fact that estrogen-promoted NPPC expression in granulosa cells can be enhanced by interaction with FSH (Lee et al., 2013). However, the oocytes within antral follicles did not show precocious resumption of meiosis after deletion of the estrogen receptor or *Cyp19α1* (aromatase) (Krege et al., 1998; Dupont et al., 2000; Kiyama and Wada-Kiyama, 2015), possibly implying that there are other pathways mediating NPPC/NPR2 action. In line with this speculation, we have proved that the expression of the NPPC/NPR2 system in ovarian granulosa cells is up regulated by sex hormones, such as androgen and estrogen through respective hormone receptors (AR and ER) in physiological conditions, in polycystic ovary syndrome (PCOS) in mice ovaries, and in *in vitro* cultured granulosa cell lines (Liu et al., 2017; Reis and Honorato-Sampaio, 2018; Wang et al., 2018). Therefore, NPPC/NPR2 as a specific pathway potentially helps to explain the mechanism of the ovulatory disruption in PCOS (Reis and Honorato-Sampaio, 2018). In addition, Yang et al. (2019) proved that transforming growth factor β (TGF-β) could regulate the expression of NPPC in MGCs and oocyte maturation. In the presence of FSH, TGF-β further increased NPPC levels and inhibited the oocyte meiotic resumption of COCs (Yang et al., 2019). Interestingly, supplementary natriuretic peptide precursor type B (NPPB) and NPPC are effective at improving the developmental competence of oocytes recovered from small-sized antral follicles of porcine *in vitro* (Zhang W. et al., 2015; Zhang Y. et al., 2017).

**FIGURE 1:**
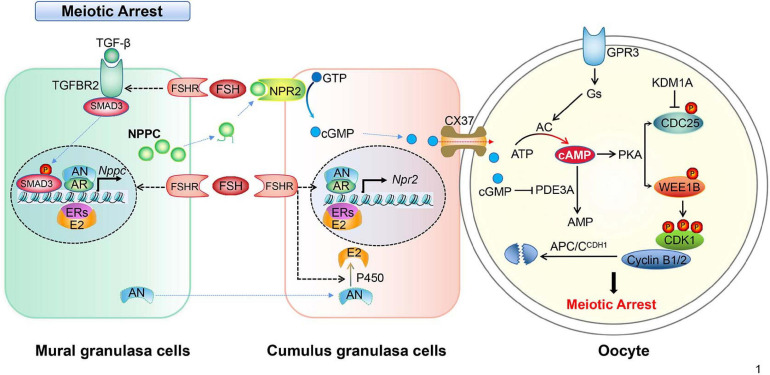
Schematic model depicting the mechanisms of meiotic arrest. Meiotic arrest in fully grown oocytes is required by the synthesis and maintenance of high levels of cAMP, the arrest state is maintained by the cooperation of granulosa cells and oocytes in the follicles. In mural granulosa cells, FSH binds its receptor (FSHR), collaborating with androgen/AR, estrogen/ER, and the TGF-β/TGFBR2 signal pathway to promote *NPPC* transcription and increase NPPC production. In cumulus granulosa cells, FSH binds FSHR, collaborating with androgen/AR and estrogen/ER to promote *NPR2* transcription and increase NPR2 production. NPPC actives NPR2, GTP is converted into cGMP, then cGMP enters the oocyte through CX37 gap junctions. In oocytes, cGMP inhibits PDE3A activity, prevents the degradation of cAMP, cAMP activates protein kinase A (PKA) that in turn activates the WEE1B kinase and inhibits the CDC25B phosphatase leading to the inactivation of CDK1. In addition, CDC25B protein level is inhibited by histone lysine demethylases 1A (KDM1A). The constant degradation of cyclin B1/2 (cycB1/2) by APC/CDH1 prevents MPF activation in the arrested oocytes.

Importantly, one of the important roles of LH surge is to downregulate the level of the NPPC/NPR2 system in MGCs and CGCs as well. The levels of NPPC/NPR2, as well as the activity of NPR2, are either completely decreased or inhibited in mouse and human ovaries after the activation of LH receptors, which occurs sufficiently earlier than GVBD. The underlined mechanism could be that LH significantly decreases AR and ER levels, and thus decreases NPPC/NPR2 levels and induces oocyte maturation (Liu et al., 2017; Wang et al., 2018; Yang et al., 2019). By suppressing the NPPC/NPR2 system, LH reduces cGMP level in CGCs as well as oocytes rapidly. Besides, the reduced cGMP level in oocytes releases PDE3A from the inhibitory state. As a result, cAMP is degraded and the maturation promoting factor (MPF) is activated, which induces the resumption of meiosis (Norris et al., 2009). However, it remains unclear how LH and FSH specifically regulate the expression of AR, ER, and TGF-β. The regulations of granulosa cells cooperate with oocytes to maintain oocyte meiotic arrest in mice, which are summarized in Figure 1.

### Oocyte Meiotic Resumption

Fully grown oocytes in early antral and preovulatory follicles have the capability to resume meiosis before LH surge (Holt et al., 2010). According to the hypothalamus-pituitary-ovary axis feedback theory, an LH surge in response to a peak estrogen surge initiates oocytes meiosis *in vivo* through positive feedback regulation. LH surge produces rapid changes in MGCs via intracellular pathways and extracellular paracrine loops. In brief, LH binds to the LH receptor (LHR) located in the membrane of theca cells and the MGCs of a follicle. As a result, the activated LHR induces serial affairs in follicular granulosa cells and oocytes. It reduces the cAMP level within the oocyte through downregulating the NPPC/NPR2 system and shutting down gap junctions between the oocyte and CGCs (Egbert et al., 2014; Shuhaibar et al., 2016). Also, it upregulates the activity of the epidermal-like growth factors (EGF) network in MGCs/CGCs (Conti et al., 2012; Jaffe and Egbert, 2017). In the oocyte, reduced cAMP levels activates the activity of MPF, which in turn phosphorylates proteins including APC and initiates GVBD and chromosome segregation (Adhikari and Liu, 2014).

#### MPF Activity Regulates the Meiotic Resumption in Oocytes

The mechanism of how high levels of cAMP are necessary to prevent meiotic maturation in oocytes is more or less fully understood. It is clear that cAMP exerts its role by activating protein kinase A (PKA). PKA balances the activities of WEE1B/MYT1 kinase and CDC25 phosphatase, and thus regulates the activity of cyclin-dependent kinase 1 (CDK1). Briefly, the CDK1 and cyclin B complex, namely MPF, is essential for oocytes meiotic maturation (Jaffe and Egbert, 2017). The ability of CDK1 to phosphorylate target proteins at specific serine and threonine residues depends on its activity and binding with the cyclin B (Jones, 2004; Jaffe and Egbert, 2017). It has been found that activated CDK1 triggers CXXC-finger protein 1 (CXXC1, also known as CFP1) phosphorylation and degradation following meiotic resumption. The degradation of CFP1 ensures the absence of the SET domain containing 1 (SETD1)-CXXC1 complex from chromatin, thereby facilitating chromosome condensation during oocyte maturation. Besides, CFP1 coordinates histone H3 lysine-4 trimethylation and meiotic cell cycle progression in mouse oocytes (Sha et al., 2018). Therefore, one of the key points to initiate oocyte meiosis depends on when to activate CDK1. In arrested oocytes, a sustained high level of cAMP activates PKA, which in turn activates WEE1. WEE1 inactivates while CDC25 activates CDK1 through phosphorylates or dephosphorylates the Thr14 and Tyr15 residues of CDK1, respectively (Chen et al., 2001; Adhikari et al., 2016; Jaffe and Egbert, 2017). Thus, the activity of MPF is indirectly controlled by the level of cAMP (Jones, 2004; Han et al., 2005; Han and Conti, 2006; Kovo et al., 2006). Interestingly, epigenetic molecules, such as histone lysine demethylases KDM1A (also known as LSD1), are involved in regulating the expression of CDC25B to maintain meiotic arrest. Conditional deletion of *LSD1* in growing oocytes results in precocious resumption of meiosis and spindle and chromosomal abnormalities (Kim et al., 2015).

Synthesis and accumulation of cyclin B1 and its interaction with CDK1 have long been considered prerequisites for oocyte MPF activation as well. As part of the MPF, cyclin B1 must be constantly degraded by a multi-subunit ubiquitin E3 ligase named the anaphase promoting complex (APC) to maintain meiosis arrest (Jaffe and Egbert, 2017). During this time, the role of cadherin 1 (CDH1) is important because it is an activator of the APC (Reis et al., 2006). Before GVBD happens, cyclin B1 translocation from the cytoplasm into the nucleus is required (Marangos and Carroll, 2004; Holt et al., 2010; Jaffe and Egbert, 2017). Interestingly, *cyclin B1*-null oocytes resumed and finished meiosis I but are then arrested at the meiosis interphase when cyclin B2 is available, indicating that cyclin B2 compensates for the shortage of cyclin B1 in oocyte meiosis I (Holt et al., 2010; Li et al., 2019).

#### Gap Junction Facilitates Intercellular Communication Within Follicles

Gap junction provides a direct communication channel between cells which allow molecules smaller than 1,000 Da be transferred to the adjacent cells (Nicholson and Bruzzone, 1997; Simon and Goodenough, 1998; Arroyo et al., 2020). In mice, as many as 20 connexins (Cxs) participate in forming the channels of the gap junction. Inside a follicle, cGMP produced in CGCs diffuses into oocytes through Cx43 and Cx37 GJs and thus elevates oocyte cGMP level (Solc et al., 2010). Importantly, the closure of GJs between MGCs and CGCs and between CGCs and oocytes are targets of LH signaling (Anderson and Albertini, 1976; Gilula et al., 1978). For instance, LH inhibits Cx43 translation and breaks down GJs to prevent cAMP and cGMP diffusion into the oocyte, which results in PKA inactivation and triggers the initiation of oocyte maturation (Kalma et al., 2004; Edry et al., 2006; Sela-Abramovich et al., 2006).

Of all connexins, Cx43 and Cx37 are the most studied ones in the follicle and may possess equal importance to folliculogenesis. In mice, Cx43 is mainly expressed in gap junctions between GCs and is regulated by extracellular signal regulated kinase-1 and -2 (ERK1/ERK2) signals in response to LH surge *in vivo* (Su et al., 2002; Sela-Abramovich et al., 2005; Dekel, 2009). However, PKCε-mediated mitogen-activated protein kinase (MAPK)-dependent signals might contribute to Cx43 phosphorylation in CGCs during FSH-induced oocyte meiotic resumption *in vitro* (Cai et al., 2018). Ovaries lacking Cx43 do not proceed beyond the primary follicle stage. Also, Cx37, which is mainly expressed between the oocyte and CGCs, is essential to oocyte growth and survival, which in turn is necessary to maintain proper MGC function (Li et al., 2007; Gershon et al., 2008). In *Cx37*-knockout mice, folliculogenesis is arrested at the early antral stage and this disruption results in sterility because mutant oocytes grow slowly and cannot survive (Carabatsos et al., 2000). To examine the roles that Cx37 and Cx43 play in oogenesis, a transgenic mouse model, in which *Cx37* specifically replaced *Cx43* in growing oocytes, was made. The generations of *Cx43* transgene mice driven by zona pellucida 3 (ZP3) crossed with *Cx37*-null mice are fertile due to the restoration of oocyte–granulosa cell coupling, oocyte growth, and oocyte maturation (Li et al., 2007). Thus, despite their different properties, Cx43 may be physiologically equivalent to Cx37 in coupling oocytes with granulosa cells. Both of them are indispensable in the regulation of oocyte maturation.

#### Epidermal Growth Factor (EGF)-Related Proteins Regulate Meiosis Resumption

EGF-related proteins are a set of proteins that respond to the LH signal and promote oocyte maturation. Different epidermal-like growth factors, such as amphiregulin (AREG), epiregulin (EREG), and beta-cellulin (BTC) are expressed in MGCs and CGCs in autocrine and paracrine manners through respective EGF receptors (EGFRs) (Tsafriri et al., 2005; Conti et al., 2006; Hsieh et al., 2007). The activation of EGFR is required for oocyte meiotic resumption and cumulus cell expansion (Fan et al., 2009; Reizel et al., 2010). Studies using inhibitors and gene deletion mouse models have identified that EGFs mediate LH action through EGFR (Park et al., 2004; Ashkenazi et al., 2005; Hsieh et al., 2007). For instance, the process of oocyte maturation, cumulus expansion, and ovulation stimulated by LH are either delayed in *AREG* or blocked in *EGFR-*deficient mice (Hsieh et al., 2007). Furthermore, in granulosa cell-specific *EDFR* deleted mice, oocytes cannot resume meiosis (Hsieh et al., 2011).

How does EGF signaling regulate meiosis resumption response to LH surge? When the LH surge arrives, LH decreases NPPC/NPR2 expression levels, thereby blocking cGMP synthesis, and stimulates MGCs to secrete EGFs to activate EGFR signaling in cumulus cells, and activates phosphodiesterase 5 (PDE5) (Egbert et al., 2016; Wang et al., 2019). The activation of PDE5 suppresses the production of NPPC and closes the gap junction communication between granulosa cells (Gershon et al., 2008; Kawamura et al., 2011; Liu et al., 2014), resulting in the decrease of cGMP levels and the reduction of oocyte cAMP levels. Then, cumulus expansion and oocyte maturation starts (Wang et al., 2019; Arroyo et al., 2020). Even though the expression of EGFR and the direct effects of EGF on oocytes has been reported (Das et al., 1991; Gall et al., 2004; Vigneron et al., 2004), how LH regulates EGF in detailed molecular mechanisms remain unclear. Recently, Wang et al. (2019) found that LH surge-induced histone deacetylase 3 (HDAC3) downregulation in GCs is essential for oocyte maturation. HDAC3 in GCs is a negative regulator of EGF expression before the LH surge. HDAC3 in GCs is recruited by transcription factors, such as FOXO1, to the *AREG* promoter to suppress the expression of *AREG*. With the LH surge, the HDAC3 level decreases while histone H3K14 acetylation increases, which enables transcription factor SP1 binding to the *AREG* promoter to initiate *AREG* transcription. Moreover, granulosa cell-specific knockout of *HDAC3 in vivo* or inhibition of HDAC3 activity *in vitro* increases the proportion of the oocyte maturation independent of LH (Wang et al., 2019). Unfortunately, the mechanism of HDAC3 downregulation after the LH surge remains unclear.

**FIGURE 2:**
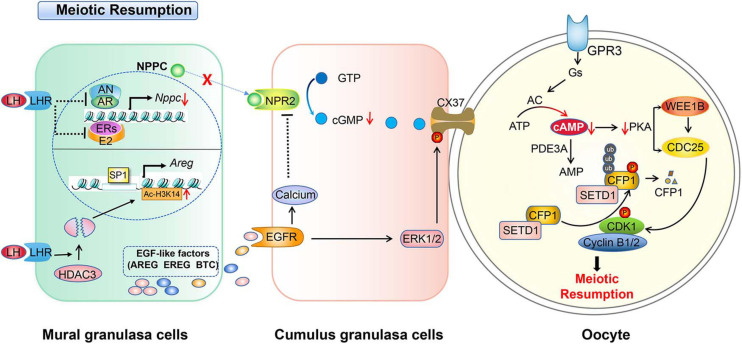
Schematic model depicting the mechanisms of LH-induced meiotic resumption. A preovulatory surge of LH binds its receptor (LHR) and induces a series of events in granulosa cells. In mural granulosa cells, on the one hand, LH/LHR inhibits AR and ER to reduce *NPPC* transcription and decrease NPPC production, on the other hand, it induces the degradation of histone deacetylase 3 (HDAC3) to decrease the Ac-H3K14 level which enables transcription factor SP1 binding to the *AREG* promoter to initiate *AREG* transcription, then increases the EGF level. The production of EGFs activates EGFR signaling and elevates the level of calcium in cumulus granulosa cells to further inactivate NPR2. LH-LHR also causes closure of gap junctions in the follicle and prevents cGMP delivery to oocytes. This in turn increases cAMP degradation by PDE3A. Low levels of cAMP and PKA can no longer activate WEE1B and inactivate CDC25B, and CDK1 becomes dephosphorylated and catalytically active. In addition, activated CDK1 triggers CXXC-finger protein 1 (CXXC1, also known as CFP1) phosphorylation and degradation following the meiotic resumption. The degradation of CFP1 ensures the absence of the SET domain containing 1 (SETD1)-CXXC1 complex from the chromatin, thereby facilitating chromosome condensation during oocyte maturation.

In addition, calcium signaling is involved in gonadotropin-induced oocyte maturation in many species (Veldhuis, 1987; Su et al., 1999; Zhang et al., 2007, 2009; Conti et al., 2012). It was reported that EGFR signaling activates phospholipase Cγ (Chattopadhyay et al., 1999), which may increase calcium levels (Wang et al., 2013). Moreover, the elevated calcium of cumulus granulosa cells inactivates NPR2, further decreasing the binding affinity of NPR2 for NPPC. As a result, cGMP levels and meiotic resumption decreases (Hao et al., 2016). The regulations of granulosa cells cooperate with oocytes to resume meiosis induced by LH surge in mice, which are summarized in Figure 2.

## Cytoplasmic Maturation

As one the key factors that heavily determines the quality of an oocyte, the cytoplasmic maturation of oocytes is critically important, which includes the synthesis, activation, and degradation of maternal mRNA as well as orderly arrangement of organelles (Schellander et al., 2007; Su et al., 2007; Walser and Lipshitz, 2011; Yu et al., 2016a), thereby affecting fertilization and embryonic development (Sirard, 2001; Chen et al., 2013; Li and Albertini, 2013; Coticchio et al., 2015; Pan et al., 2020; Sun et al., 2020; Xu et al., 2020).

### The Expression and Degradation of Maternal mRNA

The expression and degradation of maternal mRNA is developmental stage dependent. Along with the growth of activated follicles, the transcription of genes necessary for oocyte growth and meiosis resumption accumulate and are stored in the cytoplasm. With the initiation of meiotic resumption, not only does the transcription in oocytes cease because of staining agglutination, but the maternal mRNAs stored in oocytes are degraded and gradually consumed. As to protein synthesis, although a large amount of maternal mRNA exists in fully grown oocytes at the GV stage, they are translationally dormant in mice until meiotic maturation (Piccioni et al., 2005; Chen et al., 2011, 2013). The freshly translated proteins after oocytes resume meiosis play important roles in meiotic spindle assembly, MII arrest maintenance, and mRNA clearance during maternal zygotic transition (MZT) (Walser and Lipshitz, 2011). Generally, novel mRNA synthesis is initiated in the late stage of fertilized eggs (Piccioni et al., 2005).

How could the transcriptions of mRNA in growing oocytes remain stable before GVBD? Accumulative data show that there are stringent mRNA stabilizing mechanisms within GV-stage oocytes. For instance, cytoplasmic polyadenylation of the 3′-untranslated region (3′-UTR) is closely related to mRNA stability and mRNA translational activation, which plays an important role in oocyte maturation (Yang et al., 2017). Actually, the degree of polyadenylation of mRNA affects oocyte translation activation as well. Cytoplasmic polyadenylation is a key process that serves to unmask particular mRNAs and enables them to be translated (Richter, 2007; Ivshina et al., 2014). In its simplest form, masked mRNA refers to dormant transcripts in the oocyte that are to be translated during completion of meiotic divisions or in early embryos.

The degradation of maternal mRNA is controlled strictly in oocytes undergoing meiotic resumption and in early embryos. In mouse oocytes, transcriptional destruction, especially the transcripts of oxidative phosphorylation, energy production, and protein synthesis during the transition from GV to MII, is selective rather than promiscuous. It is stated that the selective degradation of the transcription of maternal mRNAs is a prerequisite for the activation of the zygotic genome (Su et al., 2007; Yu et al., 2016a,b). Particularly, regulation of maternal mRNA translation and degradation mainly occurs in maturing oocytes rather than in fertilized eggs, but these mechanisms are essential for the oocyte and zygote to build up competence to accomplish MZT.

The starting point of the MZT is oocyte activation from meiotic arrest rather than fertilization (Sha et al., 2019). About 90% of the maternal mRNA is degraded at the two-cell stage of early mice embryos (Schellander et al., 2007). In the major pathway of mRNA degradation, shortening of the poly(A) tail, or deadenylation, is the first and rate-limiting step (Walser and Lipshitz, 2011). Poly(A) tail shortening reduces the binding of poly(A) binding proteins (PABPs) and slows down translation (Ozturk and Uysal, 2017). In this aspect, prepared RNA-binding proteins (RBPs) in fully grown oocytes are important for sustaining genome stability, stabilizing and/or degrading mRNAs, or even for protein synthesis and degradation. For instance, meiosis arrest female 1 (MARF1) is an essential regulator of important oogenic processes leading to female fertility and the development of healthy offspring by suppressing levels of specific transcripts (Su et al., 2012a,b). More information about oocyte-specific RBPs regulating genome stability and mRNA stability needs to be uncovered.

### Orderly Arrangement of Organelles Is Important for a Fully Grown Oocyte

The cytoplasmic maturation of oocytes also includes the maturation of various organelles, especially cortical granules, mitochondria, the endoplasmic reticulum (ER), and cytoskeleton. The time dependent distribution and structure of these organelles are indispensable for the respective functions.

Cortical granules are membranous organelles derived from Golgi complexes, which are found in the cortex of unfertilized oocytes (Liu et al., 2003) and play important roles during the fertilization process (Liu, 2011). Following fertilization, cortical granules undergo exocytosis to release their contents into the perivitelline space, which result in the blocking of polyspermy by modifying the oocytes’ extracellular matrices, such as the zona pellucida in mammals (Coticchio et al., 2015). Besides, mitochondria are the key to ATP energy supply in oocytes. Impaired oocyte quality, including meiosis chromosome separation, maturation, and fertilization failure, correlates with both abnormal mitochondrial rearrangement and low ATP level (Blerkom, 2004; Dumollard et al., 2006). In addition, ER is responsible for the storage and release of free Ca^2+^ in the cytoplasm in oocytes, which is important for calcium reaction at fertilization (Bootman et al., 2002; FitzHarris et al., 2007; Machaca, 2007). Since the cytoskeleton is mainly composed of microtubules and filaments and the recombination of spindles is strictly controlled by the microfilament network (Verlhac et al., 2000), anything that disrupts either microtubules or microfilament causes failure of chromosome movement and separation, in which case, the oocyte is arrested at the metaphase stage.

## Epigenetic Modification Involved Systematically in Oocytes Development and Maturation

Multiple posttranslational modifications exist in developing oocytes, including acetylation, phosphorylation, methylation, glycosylation, ubiquitination, and SUMOylation of various proteins (Allfrey et al., 1964; Gutierrez and Hnilica, 1967; Huletsky et al., 1985; Kim J. H. et al., 2003; Sarmento et al., 2004; Nathan et al., 2006; Koprinarova and Russev, 2008; Rada-Iglesias et al., 2009; Xu et al., 2009), implying that epigenetic modification plays different but important roles during the oocyte maturation process under varying temporal and environmental conditions (Li, 2002; Richardson, 2002). As follows, the changes and regulation as well as functions of histone modifications during meiotic maturation of mammalian oocytes, with particular emphasis on histone acetylation and methylation are summarized.

### Histone Acetylation Modifications

Lysine acetylation of histones is generally controlled by histone acetyl transferases (HATs) and histone deacetylases (HDACs) (Gallinari et al., 2007). Acetylation of H3/4 leads to open chromatin configuration, enhances transcriptional activity, and thereby promotes transcription factor binding to DNA (Liu et al., 2011). Contrarily, deacetylation is associated with transcriptional inactivation. The key sites of histones for acetylation include at least four conserved lysines (K) in histone H4 (K5, K8, K12, and K16) and two conserved lysines (K) in H3 (K9 and K14) (Bjerling et al., 2002; de Ruijter et al., 2003). In general, all lysine residues are acetylated in fully grown GV oocytes, including H4K5ac, H4K8ac, H4K12ac, H4K16ac, H3K9ac, and H3K14ac, except for H4K8ac, which is deacetylated in condensed chromosomes and is maintained until the MII stage (Kim J. M. et al., 2003; Akiyama et al., 2004; Kageyama et al., 2007). In mammals, as many as 18 HDACs are identified and divided into four classes based on their homology with yeast proteins (Bolden et al., 2006). In which, class I HDACs are nuclear-localized, including HDAC 1, 2, 3, and 8 (de Ruijter et al., 2003; Ropero and Esteller, 2007). Class II is divided into IIa (HDAC 4, 5, 7, and 9) and IIb (HDAC 6 and 10), both of which can shuttle between the nucleus and cytoplasm (de Ruijter et al., 2003). Class III includes seven sirtuins (SIRT1-7), which are homologous with the yeast SIRT2 family proteins and require NAD^+^ as a cofactor to function (Sengupta and Seto, 2004). HDAC11 is the only member of class IV, which is homologous to both classes I and II (Gao et al., 2002). The respective actions of these proteins in oocytes are reviewed in the following.

#### Class I HDACs

Class I HDACs are important in oocyte development and maturation. HDAC1 and HDAC2 share high amino acid homology and work together in almost all repressive transcriptional complexes (Grozinger and Schreiber, 2002). HDAC1 and HDAC2 are located in the nucleus throughout oocyte growth (Ma et al., 2012). HDAC1 in the nucleus decreases gradually during the growth of oocytes and co-localizes with chromosomes following meiosis resumption. In contrast, HDAC2 in the nucleus increases between 5 and 12 days post-partum, and is relatively stable during the growing period of mice oocytes. After GVBD, HDAC2 in an oocyte is uniformly dispersed throughout the cytoplasm (Ma and Schultz, 2008; Ma et al., 2012). Germ-line deletion of either *HDAC1* or *HDAC2* will cause mouse embryo lethality (Montgomery et al., 2007; Leboeuf et al., 2010). However, conditional knockout *HDAC1* by *ZP3*-Cre has no obvious impact on fertility and oocyte maturation. Although, the deletion of *HDAC2* by *ZP3*-Cre did result in reduced fertility, but the follicular development was normal. Further, deletion of both *HDAC1* and *HDAC2*, however, results in infertility due to oocyte development arrest at the secondary follicle stage (Ma et al., 2012). The low level acetylation of H4K16 is essential for the function of centromeres. Interestingly, the deletion of maternal *HDAC2* caused high level acetylation of H4K16 and resulted in disorder in chromosome segregation and kinetochore function during MII in oocytes (Ma and Schultz, 2013). In summary, HDAC1/2 regulate oocyte growth with their compensatory function, and HDAC2 could be more critical than HDAC1 for oogenesis.

HDAC3 is expressed in the nucleus of GV oocytes and disperses in the cytoplasm of oocytes after meiotic resumption. The signal of HDAC3 accumulates on the meiotic spindle region from pre-metaphase I to MII. Knockdown of *HDAC3* in oocytes results in spindle/chromosome organization failure, with severely impaired kinetochore-microtubule attachments. In addition, overexpression of *HDAC3* modulates the acetylation status of α-tubulin in mouse oocytes (Li et al., 2017). HDAC3 also has functions in promoting meiotic apparatus assembly in aging mouse oocytes. Overexpression of *HDAC3* in old oocytes not only partially prevents spindle/chromosome disorganization, but significantly lowers the incidence of aneuploidy (He et al., 2019). HDAC3 also plays important roles in GCs. Conditional knockout of *HDAC3* in MGCs *in vivo* or inhibition of HDAC3 activity *in vitro* promotes the maturation of oocytes independent of LH (Wang et al., 2019). The above results indicate that HDAC3 in both granulosa cells and oocytes plays important regulatory roles in oocyte maturation.

HDAC8 could be important for oocyte maturation according to its distribution in growing oocytes. It is widely distributed in the cytoplasm of mouse oocytes at the GV stage. After GVBD, it starts to accumulate around the chromosomes, and shows a spindle pole-like localization pattern in both MI and MII. Inhibition of HDAC8 in fully grown oocytes causes spindle defects and chromosome misalignment during oocyte meiotic maturation, accompanied by impaired kinetochore-microtubule attachments (Zhang K. et al., 2017). Conditional deletion of *HDAC8* by *Vasa*-Cre results in subfertile females, which is independent of chromosome segregation errors during meiosis (Vijay Pratap et al., 2019). On the whole, HDAC8 is important for oocyte development and maturation, but the mechanisms of its action on oocytes needs further study.

#### Class IIa

The function of class IIa HDACs in oocyte maturation has not been well studied. According to existing reports, the expression of HDAC4 is maintained at a high level in fully grown oocytes until the MII stage, and then dramatically decreased after fertilization, it may play specific roles during mouse oocyte maturation (Kageyama et al., 2006).

#### Class IIb

In class IIb, HDAC6 has been studied extensively, while HDAC10 has hardly been reported. HDAC6 localizes in the cytoplasm of mouse GV oocytes. Overexpression of *HDAC6* results in GV oocytes and pronuclear zygotes which results in altered nuclear structure and causes compaction of the chromatin (Verdel et al., 2003). In addition, inhibition of HDAC6 in GV oocytes prevents PB1 extrusion later because of disrupted maturational progression and meiotic apparatus assembly (Zhou et al., 2017; Sui et al., 2020a). However, *HDAC6* KO mice are viable and fertile and presented no major observable phenotype (Zhang et al., 2008). Despite that, the TuA-treated group presented significant changes in the expression of *HDAC* subfamily genes such as *HDAC6*, *10*, and *11* and *sirtuin 2*, *5*, *6*, and *7* by RNA-sequencing, which may indicate that TuA is a multifunctional inhibitor which targets both HDAC and sirtuin activity rather than being a HDAC6-specific inhibitor in mouse oocytes (Choi et al., 2019).

#### Class III

Sirtuins are generally important for oocyte development. SIRT1, SIRT2, SIRT3, and SIRT6 are beneficial for improving the competence of oocytes grown or matured *in vitro* in humans and animals (Tatone et al., 2018). SIRT4, SIRT5, and SIRT7 have seldom been studied so far. Activation of SIRT1 by resveratrol *in vitro* improves oocyte quality and embryo development in mice, pigs, and cows (Liu et al., 2013; Takeo et al., 2014; Wang et al., 2014; Itami et al., 2015; Li et al., 2016; Khan et al., 2017). SIRT1 relates to mitochondria biosynthesis and degradation in oocytes because resveratrol supplementation improves the mitochondrial function and the developmental capability of the oocytes (Sato et al., 2014). In contrast, specifically inhibition of SIRT1 results in increased ROS production and abnormal MII plates in mouse oocytes (Di Emidio et al., 2014). Similarly, inhibition of SIRT2 during *in vitro* oocyte maturation or knockout of *SIRT2* blocks the progression of oocyte development after GVBD (Riepsamen et al., 2015). *SIRT2* knockdown also affects spindle organization and chromosome alignment during meiosis (Zhang L. et al., 2014). Besides, SIRT3 regulates the ROS level in oocytes. Overexpression of *SIRT3* reduces the spindle defects and chromosome misalignment in oocytes (Zhang L. et al., 2015). Last, SIRT6 is important in regulating meiotic progression as well. Depleted *SIRT6* results in disruption of spindle morphology and chromosome alignment in oocytes (Han et al., 2015).

#### Class IV

The expression of HDAC11 in oocytes decreases from the GV to MII stage. Inhibition of HDAC11 by JB3-22 significantly interrupted mouse oocyte meiosis progress, possibly because of abnormal spindle organization and misaligned chromosomes, impaired kinetochore-microtubule attachment, and spindle assembly checkpoint function (Sui et al., 2020b).

The function of HDACs during oocyte maturation are summarized in Table 1.

**TABLE 1:** Role of HDAC in oocyte development and maturation.

Protein (gene)	Phenotype *in vitro*	Phenotype of KO	Phenotype of cKO	References
HDAC1	–	Embryo lethality between E9.5-E10.5	*Hdac1*^*ZP3–Cre*^: no phenotype	Leboeuf et al., 2010; Montgomery et al., 2007; Ma et al., 2012
			*Hdac1*/*Hdac2*^*ZP3–Cre*^: infertility due to oocyte development arresting at the secondary follicle stage	
HDAC2	–	Embryonic and postnatal lethality	*Hdac2*^*ZP3–Cre*^: reduced fertility but the follicular development is normal	
HDAC3	Knock down of *Hdac3* in oocytes caused pindle/chromo some organization failure.	Embryonic death at or around the time of gastrulation	*Hdac3*^*Foxl2–ERT2–Cre*^: promotes the maturation of oocytes independent of LH	Bhaskara et al., 2008; Li et al., 2017; Wang et al., 2019
	Using HDAC3 inhibitor promotes the maturation of oocytes independent of LH			
HDAC6	Inhibition by TubA: the maturational progression and meiotic apparatus assembly in mouse oocytes, and the oocytes failed to extrude the first polar body	Viable and fertile	–	Zhang et al., 2008; Bobrowska et al., 2011; Zhou et al., 2017; Sui et al., 2020a
HDAC8	knockdown by si-RNA or drug inhibition with its selective inhibitor PCI-34051: spindle defects and chromosome misalignment during oocyte meiotic maturation, accompanied by impaired kinetochore-microtubule attachments	Death within 4–6 h of birth from brain hemorrhaging	*Hdac8^*Vasa–Cre*^:* females were subfertile. *Hdac8*^*ZP3–Cre*^: oogenesis and folliculogenesis appeared normal and mice were fertile.	Zhang K. et al., 2017; Vijay Pratap et al., 2019
HDAC11	Inhibition of HDAC11 with its selective inhibitor JB3-22: interrupted mouse oocytes meiosis progress, abnormal spindle organization and misaligned chromosomes, impaired kinetochore-microtubule attachment and spindle assembly checkpoint function	Viable	–	Cheng et al., 2014; Sui et al., 2020b
SIRT1	Activation of SIRT1 by resveratrol *in vitro* improves oocyte quality and embryo development. Inhibition of SIRT1 results in increased ROS production and abnormal MII plates in mouse oocytes	Embryonic and fetal lethality	–	Liu et al., 2013; Di Emidio et al., 2014; Takeo et al., 2014; Wang et al., 2014; Itami et al., 2015; Li et al., 2016; Khan et al., 2017
SIRT2	Inhibitor and Knockdown: the progression of oocyte development was blocked	Viable	–	Zhang L. et al., 2014; Riepsamen et al., 2015
SIRT3	Overexpression of *Sirt3* reduces the spindle defects and chromosome misalignment in oocytes	Viable	–	Lombard et al., 2007; Zhang L. et al., 2015
SIRT6	Depleted *Sirt6* results in disruption of spindle morphology and chromosome alignment in oocytes	Postnatal lethality	–	Mostoslavsky et al., 2006; Han et al., 2015

#### Histone Acetyl Transferases (HATs)

Histone acetyl transferases, including MYST, GCNA5/PCAF, and p300/CREB-binding protein (CBP), regulate the acetylation of histones as well (Gu et al., 2010).

MYST is an acronym of its four founding members, including human MOZ (monocytic leukemia zinc finger protein), yeast Ybf2 (renamed Sas3, for something about silencing 3), yeast Sas2, and mammalian TIP60 (HIV Tat-interacting 60 kDa protein) (Carrozza et al., 2003; Yang, 2004; Thomas and Voss, 2007). Importantly, K (lysine) acetyltransferase 8 (KAT8) is a highly conserved MYST family member who is specifically responsible for H4K16 acetylation and is important for mouse oocyte development, by regulating reactive oxygen species levels (Thomas et al., 2007; Gupta et al., 2013; Yin et al., 2017). The expression of *KAT8* increases dramatically between 14 days and full-grown GV-stage oocytes, followed by a sharp decrease in GVBD-MI-stage oocytes. The protein is mainly located in the nucleus throughout the growth phase, but upon GVBD, the staining intensity decreases and the signal becomes uniformly dispersed throughout the oocyte. Oocyte *KAT8* deletion results in female infertility with defects in follicle development and increased oocyte apoptosis (Yin et al., 2017).

In bovine, the levels of MYST4 mRNA in both GV and MII oocytes are high. MYST4 protein accumulates in the nucleus of GV oocytes. It concentrates in the vicinity of the meiotic spindle rather than on chromosomes in the MI stage (Champagne et al., 1999; McGraw et al., 2007).

Histone acetyltransferases p300 and the CBP subfamily are constitutively expressed in the GCs of growing and ovulatory follicles in a gonadotrophin-independent manner. ED-rich tail (CITED) protein CITED4 formed an endogenous protein complex with CBP and transcription factors CCAAT/enhancer binding protein C/EBP/b, which docked on the promoters of LH and ERK1/2 target genes. Both CITED4 expression and CBP acetyltransferase activity were indispensable for ovulation-related molecular and histological events. Moreover, the dynamic histone acetylation changes (histone H2B-Lys5 and H3-Lys9) in GCs were regulated by LH, CBP, and HDACs during ovulation (Zhang Y. L. et al., 2014). In addition to the above two subfamilies, the constant expression of *HAT1* and *GCN5* mRNA was also detected during bovine oocyte maturation (McGraw et al., 2003).

Taken together, HATs have essential roles in mouse follicle development and oocyte maturation, and the potential functions of HATs in oocytes maturation needs more exploration.

### Histone Methylation Modifications

Histone methylation correlates with chromatins activity. For instance, H3K4 methylation is associated with the activation of chromatins and occurs mainly in the promoter regions of active genes, while the methylations of either H3K9 or H3K27 relates to gene inactivation (Werner and Ruthenburg, 2011). In developing mouse oocytes, the level of H3K4me3 are elevated during the transition of chromatin configuration from the non-surrounded nucleolus (NSN) to surrounded nucleolus (SN) type (Yu et al., 2017), the latter of which have better developmental competence after fertilization (Ma et al., 2013; Zhang et al., 2019). H3K4me and H3K4me2 levels elevate, but H3K4me3 level decreases after GVBD and reaches its lowest point in anaphase I (Sha et al., 2018).

Histone methylation modification is regulated by histone lysine methyl transferases (KMTs) and histone lysine demethylases (KDMs) via modifying lysine residues and the number of methyl groups (Sha et al., 2020). There are six known histone H3 methyltransferases, including SET domain containing 1A/B (SETD1A/B), lysine (K) methyltransferase 2A/B (KMT2A/B), and KMT3/4 in mammals (Shilatifard, 2012).

The SETD1/COMPASS histone methyltransferase complex is the primary enzyme that methylates histone H3K4 by recognizing its basic subunit, CXXC1 (Roguev et al., 2001; Lee and Skalnik, 2005; Brown et al., 2017). The expression of *SETD1A* and *SETD2B* persists from oocyte to blastocyst. *SETD1A* is first required at the epiblast stage, whereas *SETD1B* becomes essential after gastrulation (Bledau et al., 2014). In *GDF9*-Cre-driven *SETD1B* deficient mice, the number of follicles decreases gradually with time, the ovulated MII oocytes exhibit meiotic spindle abnormalities. And the oocytes as well as zygotes display perturbed cytoplasmic organelles and aggregated lipid droplets (Brici et al., 2017). SETD1-CXXC1 conjugation regulates H3K4me3 in mice oocytes (Yu et al., 2017). A stabilized CXXC1 in fully grown GV oocytes resulted in decreased GVBD and PB1 emission rates as well as spindle assembly defects in mice (Sha et al., 2018).

KMT2B activates gene expression by regulating H3K4me3 (Glaser et al., 2006). Loss of *KMT2B* in mouse oocytes induced by *GDF9*-Cre resulted in abnormal meiosis maturation, anovulation, oocyte death, and female sterility, in which H3K4 level decreased and gene expression was abnormal (Andreu-Vieyra et al., 2010; Hanna et al., 2018). KDMs consist of KDM1 and the KDM2-KDM7 subfamily, which contain a Jumonji C (JmjC) domain (Xhabija and Kidder, 2019). KDM1A is expressed in the oocyte nucleus, which specifically catalyzes the demethylation of H3K4me1 and H3K4me2. *KDM1A*-null oocytes display defects in maintaining prophase I arrest and undergo precocious GVBD. Most *KDM1A*-null oocytes undergo apoptosis before the completion of meiotic maturation (Kim et al., 2015). KDM1B is highly expressed in growing oocytes and the level persists through later stages of oogenesis, but it is hardly detectible in oocytes of primordial and primary follicles in mice. The deletion of *KDM1B* in mice does not affect embryo development, animal survival, or oocyte growth. However, oocytes from *KDM1B-*deleted females show high levels of H3K4 methylation and fail-to-deposit DNA methylation marks at four out of seven imprinted genes. Early embryos derived from these oocytes show biallelic expression or suppression of the affected genes and died before mid-gestation (Ciccone et al., 2009).

KDM4A and KDM4B are located in human oocytes, granulosa cells, theca cells, and luteal cells in reproductive-aged women (Krieg et al., 2018). Deletion of KDM4A during oogenesis had no significant impact on ovulation since *KDM4A*^–/–^ and wild-type females ovulated similar numbers of fertilizable oocytes. MII oocytes from *KDM4A*^–/–^ females were euploid with no evidence of major chromosomal breakages or aneuploidies. KDM4A is the major demethylase functional in MII oocytes and is required to maintain the genomic stability of pre-implantation embryos (Sankar et al., 2020).

The KDM5 family consists of KDM5A to KDM5D (Xhabija and Kidder, 2019). During knockout of *KDM5A*, the mice are viable and fertile (Klose et al., 2007), whereas knockout of *KDM5B* resulted in early embryonic lethality (Catchpole et al., 2011), suggesting that *KDM5B* is the major functional KDM family member *in vivo*.

### Histone Phosphorylation Modifications

Protein phosphorylation occurs most often on serine, threonine, or tyrosine residues and competently regulates cell cycle stage-related affairs in a variety of different signal transduction pathways (Schatten and Sun, 2014). For example, histone H3 phosphorylation at Ser10 and Ser28 affects chromatin condensation of either mitosis or meiosis (Bradbury et al., 1973; Bradbury, 1992; Wei et al., 1998, 1999; Goto et al., 1999; Houben et al., 2005; Swain et al., 2007). Aurora B phosphorylates histone H3 at Ser28 in mitotic cells and Ser10 and Ser28 in meiosis cell. ZM447439 (an inhibitor of the Aurora kinase family) treatment prevented Aurora B activity and significantly decreased the phosphorylation levels of both H3/Ser10 and H3/Ser28 in mouse oocytes, resulting in chromosome misalignment (Jeliìnkovaì and Kubelka, 2006). In addition, protein phosphatase 1 (PP1) dephosphorylates H3 at Ser10 in budding yeast and nematodes. Inhibition of PP1/PP2a induces rapid chromosome condensation with hyperphosphorylated histone H3 (Bui et al., 2004; Swain et al., 2007). Taken together, the balance of Aurora B kinase and PP1 activities regulate the meiotic phosphorylation of histone H3 in mammalian oocytes.

### Ubiquitination Modifications

The ubiquitination/deubiquitination system is important for the degradation of proteins, cell cycle progression, and transcriptional regulation (Bassermann et al., 2014), which is also important for oocyte maturation (Dekel, 2005; Susor et al., 2010; Mtango et al., 2012). Importantly, APC initiates the metaphase to anaphase transition by inducing the degradation of cyclin B and securin (Jones, 2011). Also, protein ubiquitin (Ub) E3 ligases trigger specific protein degradation and thus plays an important role in the process of both the meiotic and mitotic cell cycle (Huo et al., 2004).

Interestingly, cullin ring-finger ubiquitin ligase 4 (CRL4) is one of E3 ligase members who exert multiple functions in the maintenance of oocyte survival and meiotic cell cycle progression (Jones, 2011). DCAF13, a CRL4 adaptor, stimulates the meiotic resumption-coupled activation of protein synthesis in oocytes. Deletion of *DCAF13* in oocytes resulted in not only decreased CDK1 activity and impaired meiotic cell cycle progression as well as chromosome condensation defects, but also polyubiquitination and degradation of PTEN (Zhang et al., 2020).

In addition, protein-ubiquitination mediated CCNB1 and securin degradation is essential for the metaphase to anterograde transition during oocyte meiotic maturation (Herbert et al., 2003; Marangos and Carroll, 2008).

Ubiquitin C-terminal hydrolases (UCHs) are a deubiquitin enzyme that catalyzes the hydrolysis of peptides, isopeptides, or UB portions (Kim J. H. et al., 2003; Wilkinson, 2009). UCHs present in oocytes in many species. UCHs have a complimentary distribution in porcine, bovine, and murine oocytes. UCHL1, one of the most abundant proteins in mammalian oocytes, accumulates in the oocyte cortex. UCHL3 is associated with oocyte spindle (Mtango et al., 2012). Inhibiting UCH activity causes excessively large PB1, distorts the meiotic spindle, and disrupts other spindle attributes, such as chromosome alignment (Mtango et al., 2012). *In vitro*, inhibition of UCHL3 reduces the expansion of cumulus cells (Mtango et al., 2014).

In follicular granulosa cells, the ubiquitin-proteasome system (UPS) was involved in regulating the deposition of the extracellular matrix of cumulus and steroidal formation during the expansion of cumulus cells, implying that this system may be pivotal for follicle development (Nagyova et al., 2012).

### Histone Glycosylation Modifications

In the female reproductive system, a large number of proteins, including FSH, LH, GDF9, BMP15, and AMH are glycosylated (Saito et al., 2008; Bousfield and Harvey, 2019). Protein glycosylation is one of the most frequent post-translational modifications (PTMs), which affects many things, such as protein folding, distribution, stability, and activity. There are two main types of glycosylation in cells, N-linked and O-linked glycosylation (Ohtsubo and Marth, 2006). Defects in the process of protein glycosylation leads to many clinical diseases.

Protein *N*-glycosylation in oocytes is crucial for female fertility. For example, DPAGT1 is an enzyme involved in the process of protein *N*-glycosylation. *DPAGT1* missense mutation causes subfertility in females due to defective follicular development and less ovulation (Li et al., 2020). Also, due to the decreased glycosylation of ZP proteins, the mutant oocytes have a thin and fragile ZP layer and have poor developmental ability after *in vitro* fertilization. Furthermore, the first meiotic division is accelerated in such mutant oocytes. Importantly, the phenotypes of conditional knockout of *DPAGT1* in infertile mouse oocytes is consistent with those in humans (Li et al., 2020).

Protein *O*-glycosylation plays a small role in oocyte maturation. *In vitro*, *O*-glycosylation is elevated in bovine COCs exposed to glucosamine (Sutton-McDowall et al., 2006). Glucosamine treatment during *in vitro* maturation does not affect the meiotic maturation of cow, pig, or mouse oocytes, but blastocyst development was severely inhibited (Schelbach et al., 2010). This suggests that protein *O*-glycosylation does not affect oocyte maturation, but it affects the quality of oocytes.

### SUMOylation Modifications

SUMOylation and de-SUMOylation modification refers to the reversible addition and removal of SUMO (small ubiquitin-related modifier) polypeptides on lysine residues (Schatten and Sun, 2014). SUMO proteins, such as SUMO1, SUMO2, SUMO3, and UBE2I, are expressed in and are required for oocyte maturation in events like oocyte meiotic resumption and spindle formation (Ihara et al., 2008; Wang et al., 2010; Feitosa et al., 2018; Rodriguez et al., 2019).

The localization of SUMO1, SUMO2, and SUMO3 in oocytes depends on the developmental stage of the oocytes. In immature oocytes, SUMO1 localizes to the nuclear membrane while SUMO2/3 are within the nucleoplasm. During oocyte meiosis, SUMO1 localizes to the spindle poles and around the chromosomes whereas SUMO2/3 locate near the centromeres (Yuan et al., 2014). UBE2I primarily expresses in the nucleoplasm of mouse growing oocytes at least from postnatal day 13 to GV-stage fully grown oocytes. UBE2I is downregulated following meiotic resumption (Ihara et al., 2008).

The importance of SUMOylation on oocytes maturation could be highlighted by the following facts. After endogenous SUMO1 or UBC9 activities in oocytes were either inhibited or silenced, the percentage of GVBD and PB1 extrusion was significantly reduced, together with abnormal spindle organization, chromosome misalignment, segregation defects, and aneuploidy in matured oocytes (Yuan et al., 2014). Similarly, inhibition of UBE2I for GV-stage mouse oocytes disrupts meiotic maturation and causes defects in spindle organization (Yuan et al., 2014), while overexpression of *UBE2I* in meiotic incompetent oocytes stimulates gene transcription *in vitro* (Ihara et al., 2008). Deletion of UBE21 by *GDF9*-Cre results in complex infertility phenotypes, including defects appearing at multiple critical oocyte transition points, such as unstable ovarian reserves, impaired communication with granulosa cells, and defective resumption of meiosis and meiotic progression (Rodriguez et al., 2019).

Other proteins that affect oocyte maturation are also involved in the process of SUMOylation. For instances, overexpression of the SUMO-specific isopeptidase, sentrin/SUMO-specific protease 2 (SENP2), leads to defects in MII spindle organization by changing the localization of SUMO-modified proteins in oocytes (Wang et al., 2010). Septin, a conserved GTP-binding protein that is modified by SUMO1, is also required for chromosome congression in mouse oocytes (Zhu et al., 2010). And the spindle-assembly checkpoint protein Bub1-related kinase, or MAD3/Bub1b (BUBR1), may be SUMOylated by SUMO1 and is necessary for homologous chromosome alignment as well (Wei et al., 2010; Yang et al., 2012b).

## Conclusion

Oocyte maturation is a complex process involving multiple steps and is regulated by many molecules and signaling pathways. In recent years, due to the rapid development and popularization of technologies like the genetic modification of animal models, molecular biology, and biochemistry, researchers have gained a better understanding of oocyte GV arrest and meiosis I resumption. The major cellular and molecular affairs, especially the epigenetic modification events related to oocyte maturation in response to hormone induction, and the major advances in this field, are highlighted in this review.

Since the development of an oocyte depends not only on the oocyte itself, but on mutual communication and physical contact with follicular granulosa cells, it is important to focus more on epigenetic changes within oocytes, ovarian granulosa cells in response to hormones, and other extracellular molecules induction. Besides, applying microscopes with high resolution and a high-throughput analysis technique, such as mono-cellular based sequencing and omics techniques, should be emphasized to present clearer 3D or even time-dependent 4D representations of critical affairs that happen during oogenesis. Finding more specific oocyte-expressed proteins, such as RBPs and oocyte-derived paracrine molecules, may contribute to uncover the mysterious mechanisms of oocyte meiosis as well. Further, integration of analysis of sequencing data, comparing the data collected from different breeds, and verifying the function of each individual molecule *in vitro* and *in vivo* simultaneously based on multiple animal models are also plausible.

## Author Contributions

MH and TZ collected the information and wrote the manuscript. CW and YY revised the manuscript. All authors read and approved the final manuscript.

## Conflict of Interest

The authors declare that the research was conducted in the absence of any commercial or financial relationships that could be construed as a potential conflict of interest.
